# Postpartum Guillain-Barré Syndrome: A Case Report and Literature Review

**DOI:** 10.7759/cureus.55207

**Published:** 2024-02-29

**Authors:** Minghui Chen, Mark C Norris, Jessica H Kwan, Tao Li

**Affiliations:** 1 Anesthesiology, Boston Medical Center, Boston, USA; 2 Health Policy, Oregon State University, Corvallis, USA

**Keywords:** literature review, obstetric care, labor and delivery, case report, anesthesia, guillain-barré syndrome, postpartum

## Abstract

Guillain-Barré syndrome (GBS) is a rare acute-onset neurological disease with significant morbidity and mortality. The risk of GBS increases after delivery. Labor and delivery presents many possible risk factors for GBS. However, risk factors and prognosis of postpartum GBS remain unclear due to its low incidence. Here, we first present a patient with a history of postpartum GBS who returned for an elective repeat cesarean section (C-section). For her previous delivery, the patient received spinal anesthesia for an urgent C-section. She presented postpartum with jaw pain, facial palsy, respiratory difficulty, progressive bilateral lower extremity weakness, and areflexia. The diagnosis of GBS was confirmed by cerebrospinal fluid (CSF) examination, nerve conduction studies (NCS), and electromyography (EMG). Her symptoms of GBS improved after intravenous immunoglobulin (IVIG) treatment. The patient also had an *Escherichia coli*-positive urinary tract infection (UTI), which was treated with nitrofurantoin. For her repeat elective C-section, we performed a dural puncture epidural (DPE) anesthesia. After delivery, she was discharged to home uneventfully. She did not report any new neurological symptoms at her three-week follow-up. Here, we also review published cases of postpartum GBS and discuss peripartum anesthetic considerations for patients with GBS, aiming to inform clinical management of postpartum GBS in the future.

## Introduction

Guillain-Barré syndrome (GBS) is a rare acute-onset immune-mediated polyneuropathy that primarily features ascending flaccid weakness but can also affect sensory and autonomic peripheral nerves. The incidence of GBS in North America and Europe is about 0.81-1.91 per 100,000 person-years [1]. GBS is highly heterogeneous clinically and can be broadly classified as acute inflammatory demyelinating polyradiculoneuropathy (AIDP), acute motor axonal neuropathy (AMAN), or acute motor sensory axonal neuropathy (AMSAN). GBS is a serious disease associated with high acute phase mortality (about 5%) and significant long-term morbidity even after standard immunotherapies [1].

Labor and delivery may involve multiple possible risk factors for GBS, such as infection, immunization [1], surgery [2], preexisting comorbidities (e.g., autoimmune diseases and malignancy), and neuraxial anesthesia [3]. The incidence of GBS increases during the first 30 postpartum days [4]. However, due to the rarity of postpartum GBS, risk factors, incidence, and prognosis remain unclear. Case reports [3,5-15] and a single-center study [16] suggest that the mode of delivery, preceding infection, and first pregnancy may be associated with the onset of GBS. A retrospective single-center study reported that post-surgical GBS has a worse outcome than non-surgical GBS [17].

In this paper, we first present a unique case of a patient with a history of postpartum GBS returning for a repeat elective cesarean section (C-section), followed by a review of published cases of postpartum GBS. Our patient provided written Health Insurance Portability and Accountability Act (HIPAA) authorization for publication of this case report. This information was previously presented as a meeting abstract at the ANESTHESIOLOGY annual meeting on Oct 14, 2023.

## Case presentation

**Figure 1:**
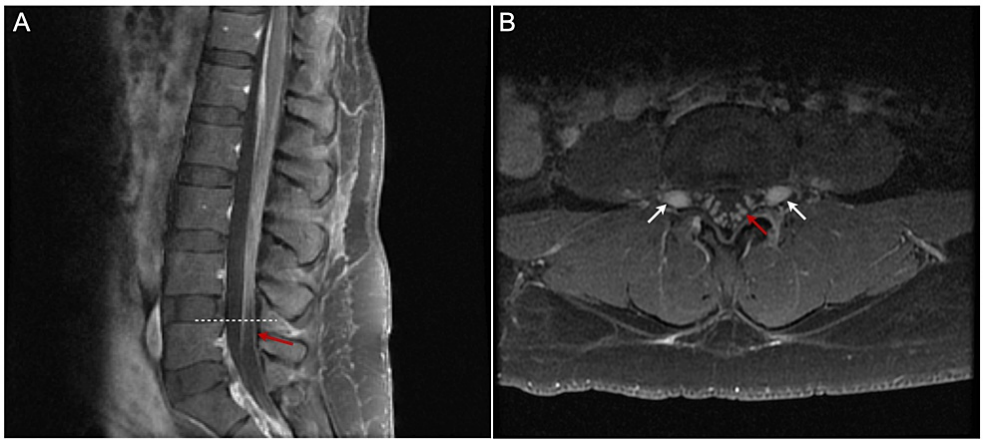
Lumbar spine magnetic resonance imaging (MRI) of a 41-year-old female with postpartum Guillain-Barré syndrome. Post-contrast fat-suppressed sagittal T1-weighted MRI (A) and axial T1-weighted MRI (B) showed mild smooth thickening and prominent enhancement of the cauda equina (red arrows in A and B) and exiting nerve roots (white arrows in B). The level of Figure 1B is shown as the dotted line in Figure 1A.

A 41-year-old female, G5P2022 at 38 weeks of gestation with gestational diabetes mellitus, presented to our institution for an elective repeat cesarean delivery for breech presentation. Her previous medical record showed that she delivered her second child via urgent C-section for failure to descend nine years ago. That pregnancy was complicated by mildly elevated blood pressure with an increased protein-to-creatinine ratio of 0.35. During that pregnancy, the patient received tetanus-diphtheria-pertussis (TDAP) vaccine in the third trimester, but no influenza vaccine. She received an uncomplicated spinal anesthetic using 0.75% bupivacaine, with 8.25% dextrose 12 mg, fentanyl 15 mcg, and preservative-free morphine 200 mcg. No infection was detected before or immediately after delivery. On the 11th day after delivery, she developed progressive numbness and weakness in her lower extremities, severe jaw pain, inability to speak, and numbness and weakness in her face. On the 26th day postpartum, she was brought to the emergency department in a wheelchair. Physical examination revealed mild fever, bilateral facial palsy, 0/5 strength in bilateral lower extremities, and no sensation of pinprick in lower extremities. Bilateral patellar and ankle-deep tendon reflexes were absent. Complete blood count revealed mild leukocytosis. Other blood tests, including erythrocyte sedimentation rate (ESR), C reactive protein (CRP), thyroid-stimulating hormone (TSH), and C3/C4 complement, were normal. Urine culture was positive for *Escherichia coli*. The diagnosis of GBS was confirmed by albuminocytologic dissociation in cerebrospinal fluid (CSF) total protein 423, albumin 312, and IgG 55.6 mg/dL; glucose 76 mg/dL; total nucleated cells 1/mm^3^, mononuclear 20%). Magnetic resonance imaging (MRI) of the lumbar spine (Figure 1) showed mild smooth thickening and prominent enhancement of the cauda equina and exiting nerve roots, without nodularity or clumping, consistent with inflammatory processes, such as GBS. MRI brain and magnetic resonance angiography (MRA) of the head and neck were normal. Nerve conduction studies (NCS) performed 2.5 weeks after the onset of illness showed an absent sensory response in the right median and ulnar nerves; absent motor response of the right median and facial/nasalis nerves; decreased motor amplitude, prolonged distal motor latencies, and slowed motor conduction velocities in right ulnar, radial, tibial, and peroneal nerves; marked temporal dispersion with increased compound muscle action potential (CMAP) duration (left median: 19.6 ms, right ulnar: 11.5 ms, tibial: 31.0 ms, peroneal: 13.3 ms); and absent F-waves in the right ulnar, median, and tibial nerves. Concentric needle electromyography (EMG) was performed in selected right upper and lower extremity muscles. Spontaneous activity in the form of fibrillation potentials and positive sharp waves was seen only in the right frontal where no voluntary motor units were seen. The results of electrophysiological studies were consistent with acute inflammatory demyelinating polyradiculoneuropathy (AIDP). During hospitalization, she developed respiratory difficulty but did not require intubation or ventilatory support. Both respiratory difficulty and limb weakness were dramatically improved after a five-day course of 0.25 g/kg/day intravenous immunoglobulin (IVIG) treatment. She also received nitrofurantoin monohydrate/macrocrystals (MACROBID) 100 mg orally twice a day for seven days for an *E. coli *urinary tract infection (UTI). At the 10-week follow-up, she still had bilateral peripheral facial nerve weakness, right more than left, with bilateral symmetric paresis of the orbicularis oculi (without Bell’s phenomenon), with frontalis 0 on the right and trace on the left, with orbicularis oris 0 on the right and 2+ on the left. Upper extremity strength was 5/5, and lower extremity strength was 4/5 for hip flexion bilaterally and 5/5 distally. Deep tendon reflex and pinprick and vibration sensation were normal.

Nine years later, the patient returned for a repeat elective C-section and bilateral tubal ligation. A neurology consult was obtained, and their preoperative neurological examination revealed mild right facial palsy with no residual limb weakness or sensory changes. The risk of recurrent GBS was thought to be low. The patient was instructed to self-monitor for any sensory/motor changes or worsening dyspnea over the two to three weeks after the C-section.

To limit spinal nerve exposure to local anesthetics, and to produce a slower onset of sympathetic blockade, we chose a dural puncture epidural (DPE) anesthesia for her surgery. A total of 0.5% ropivacaine 35 mL and fentanyl 100 mcg were injected incrementally via epidural catheter throughout the two-hour 20-minute operation. The patient tolerated the surgery well without hemodynamic instability and delivered a healthy baby. The epidural catheter was removed two hours postoperatively. No new neurological deficits were detected at discharge. At the three-week follow-up, the patient was ambulating well and reported no new neurological deficits.

## Discussion

Literature review of postpartum GBS

We performed a literature search in Medline/PubMed using the following keywords: (“Guillain-Barre syndrome” or “acute inflammatory demyelinating polyneuropathy” or “AIDP” or “acute motor axonal neuropathy” or “AMAN” or “acute motor sensory axonal neuropathy” or “AMSAN”) AND (“postpartum” or “obstetric” or “labor and delivery” or “epidural anesthesia” or “spinal anesthesia” or “epidural analgesia” or “combined spinal and epidural analgesia” or “neuraxial anesthesia”) and limit language to English. A total of 114 citations were identified, of which 44 were case reports. We excluded case reports without definitive GBS diagnosis by CSF examination and/or electrophysiologic studies, including NCS and EMG, or patients with symptoms of GBS arising before or during pregnancy.

We identified 12 cases of GBS after obstetric procedures (Table 1) [3,5-15]. Patients’ ages ranged from 16 to 34 years old. Six patients were primigravida [8-12,14], four multigravida [3,7,13,15], and two unknowns of gravidity [5,6]. Seven patients underwent C-section [5,7,9,10,13-15], two had vaginal delivery [3,12], one with a forceps-assisted vaginal delivery [8], one opted for pregnancy termination [6], and one report lacked information on the mode of delivery [11]. Among the seven patients who received C-sections, except one who received the surgery electively [13], the other six underwent an urgent or emergent procedure. Four cases employed epidural analgesia or anesthesia [3,8,9,13], two spinal anesthesia [10,14], two general anesthesia [6,7], one did not receive any form of anesthesia [12], and three cases lacked information on anesthesia type. All 12 patients initially presented with bilateral lower extremity weakness. The onset of GBS symptoms ranged from immediately after delivery up to 40 days postpartum. Five cases had antecedent infection [5,7,9,10,13], including two diagnosed with COVID-19 [5,7]. The three cases with antecedent infection other than COVID-19 had onset of GBS symptoms within 24 hours postpartum [9,10,13]. All five cases with antecedent infection delivered by C-section, two of whom required endotracheal intubation [5,10]. Based on the limited number of cases, patients with antecedent infection likely had worse outcomes compared to those without antecedent infection: one died [5]; two could not ambulate independently after 1.5 years [10] and 2 years [9]; only one recovered completely [13]; and the other one lacked long-term follow-up [7]. Six out of seven cases without antecedent infection had a later onset of GBS symptoms at least one week after delivery [3,6,11,12,14,15]. These women also had better outcomes. Four out of seven had complete motor recovery [3,8,11,14], but the other three did not have long-term follow-up. Among all 12 patients, three required intubation and mechanical ventilation [5,10,12], including one who died on the 15th day postpartum [5], one who could not ambulate independently after 1.5 years [10], and one who lacked a long-term follow-up [12].

The 12 patients received various treatments (Table 1). IVIG was administered in five cases [6,7,12,13,15], plasmapheresis was performed in five cases [5,6,8,9,11], and steroids were given in three cases [3,5,6]. One patient was treated with IVIG combined with steroids and plasmapheresis [6]. One patient was treated with steroids combined with plasmapheresis [5]. One patient did not receive any immunotherapy and recovered completely [14]. One patient does not have a record of pharmacological therapy.

**Table 1:** Summary of published cases of GBS after obstetric procedures C-section: caesarean section; UTI: upper respiratory tract infection; IVIG: intravenous immune globulin; COVID-19: coronavirus disease 2019

Age (years)	Primigravida	Anesthesia	Procedure	Antecedent infection	First presentation	Onset time	Peak time	Treatment	Outcome	Ref.
29	No	Epidural	Vaginal delivery	None	Acroparesthesias; Weakness of all four limbs; Constipation; Urgency and frequency; Dysphagia	1 week	Within 1 month	Steroids	1 year later, medication-free and asymptomatic, and neurologic examination was normal	[3]
20	Yes	Epidural	Forceps-assisted vaginal delivery	None	Left facial palsy; Acroparesthesias; Ascending weakness of all four limbs	1^st^ day	5^th^ day	Plasmapheresis	2 weeks later, patient could walk	[8]
18	Yes	Epidural	C-section (urgent)	Chorioamnionitis during labor	Lower limbs numbness and weakness; Sharp neck and upper back pain; Urinary and fecal incontinence	12 hours	5^th^ day	Plasmapheresis	7 months later, still unable to walk unassisted. 2 years later, mild balance instability remained	[9]
22	Yes	Spinal	C-section (emergent)	Diarrhea and mild fever a week ago	Lower limb weakness; Difficulty breathing	Immediate	12^th^ hour	Intubation and tracheostomy	1.5 years later, able to walk with support. 5/5 in the upper limbs and 3/5 in lower limbs	[10]
27	Yes	Unknown	Unknown	None	Lower limb weakness	40 days	47^th^ day	Plasmapheresis	3 months later, ambulation independent.	[11]
26	Yes	None	Vaginal delivery	None	Lower limb paraesthesia	14 days	17^th^ day	IVIG, Intubation	2 weeks later, extubated	[12]
29	No	Epidural	C-section (elective)	URI a week ago	Lower limb weakness; Urinary retention	Within a few hours		IVIG	2 months later, completely recovered	[13]
16	Unknown	General	Pregnancy termination	None	Lower limb paraesthesia	2 weeks	16^th^ day	Methylprednisolone, IVIG, Plasma exchange, Azathioprine		[6]
30	Unknown	Unknown	C-section (urgent for intrauterine fetal death)	COVID-19 a week before delivery	Lower limbs weakness	10^th^ day	13^th^ day	Methylprednisolone, Plasmapheresis, Intubation	Patient died on the 15^th^ day postpartum	[5]
34	No	General	C-section (urgent)	COVID-19 antepartum	Low back pain; Ascending weakness and numbness in lower limbs; Difficulty walking	9^th^ day	21^st ^day	IVIG, Enoxaparin, Pregabalin	12days later, 5/5 in upper limbs and 3/5 in lower limbs	[7]
27	Yes	Spinal	C-section (emergent)	None	Ascending weakness in bilateral upper and lower limbs	8^th^ day	22^nd^ day	Physiotherapy, Calcium, Multivitamin	1-month later, power completely improved with no sensory symptoms	[14]
27	No	Unknown	C-section (emergent)	None	Lower limbs weakness	20^th^ day	25^th^ day	IVIG		[15]

Risk factors, clinical course, and anesthesia considerations for postpartum GBS

In this article, we presented a patient with a history of postpartum GBS who subsequently had an uneventful repeat C-section under epidural anesthesia. We also reviewed 12 published cases of postpartum GBS, aiming to find possible risk factors and summarize the clinical course of postpartum GBS to facilitate early diagnosis and prompt treatment of GBS in the future.

C-section may trigger GBS [2]. Together with the case presented in this article, we found that eight out of these 13 cases underwent C-sections, seven of which were performed urgently or emergently. However, other risk factors, such as infection, may also exist in patients who require C-sections. Further retrospective studies using large-scale datasets are needed to verify whether C-section increases the risk of postpartum GBS or not.

All these 13 cases present progressive lower extremity weakness at the onset. Detection of lower extremity weakness immediately postpartum could be hindered by residual effects of neuraxial anesthesia. GBS, though rare, should be considered as a differential diagnosis in postpartum patients with worsening bilateral lower extremity weakness. Postpartum patients with ascending bilateral extremity weakness require close monitoring for bulbar weakness, respiratory insufficiency, and autonomic dysfunction. The prognosis of postpartum GBS varies from complete recovery to death. Respiratory distress and the need for mechanical ventilation are associated with worse outcomes [16]. Early diagnosis, close monitoring, and prompt treatment are important to manage postpartum GBS.

Concerns for managing a patient with a history of GBS returning for repeat C-sections include whether neuraxial anesthesia might trigger relapse and which kind of neuraxial anesthesia is safest. Although GBS is typically monophasic, recurrence of GBS has been reported. Neuraxial anesthesia has been proposed as a trigger of GBS. Postulated mechanisms include central or peripheral nerve injury due to local anesthetic exposure or direct needle injury [3]. However, this correlation is speculative and inconclusive [8,9]. Further studies are needed to elucidate this hypothesis. Two case reports of patients with preexisting GBS receiving neuraxial anesthesia demonstrated no worsening of symptoms postoperatively [18,19]. However, cardiac arrest following spinal anesthesia was reported in a GBS patient with autonomic dysfunction [20]. Because of its slower onset and lower risk of hypotension and bradycardia, epidural anesthesia may be safer than spinal anesthesia for GBS patients, particularly those with autonomic dysfunction. In addition, careful preoperative evaluation and documentation of baseline neurological status is crucial to inform decisions on appropriate anesthesia type and allow early detection of signs and symptoms suggestive of relapse. Due to the rarity of postpartum GBS, observations from case reports could be biased and are not conclusive. Future studies with large-scale datasets may further elucidate risk factors for postpartum GBS and help determine the preferred anesthesia type for patients with existing GBS.

## Conclusions

GBS is a rare acute neurological condition with high morbidity and mortality. GBS should be part of the differential diagnosis in postpartum patients presenting with progressive lower extremity weakness. For patients with preexisting GBS, detailed preoperative neurological exams and close monitoring following delivery are critical for early detection of relapse. Early diagnosis and prompt treatment are key to improving clinical outcomes. Due to the low incidence of postpartum GBS, the role of neuraxial anesthesia as a risk factor is unclear. Given the slower onset and lower risk of hypotension and bradycardia, epidural anesthesia may be safer than spinal anesthesia for patients with GBS and autonomic dysfunction.
